# Reflections on 40 Years of AIDS

**DOI:** 10.3201/eid2706.210284

**Published:** 2021-06

**Authors:** Kevin M. De Cock, Harold W. Jaffe, James W. Curran

**Affiliations:** Centers for Disease Control and Prevention, Atlanta, Georgia, USA (K.M. De Cock, H.W. Jaffe);; Emory University and Emory Center for AIDS Research, Atlanta (J.W. Curran)

**Keywords:** HIV, AIDS, HIV/AIDS, human immunodeficiency virus, acquired immune deficiency syndrome, Ebola, coronavirus disease, COVID-19, forty years, history, health security, SDGs

## Abstract

June 2021 marks the 40th anniversary of the first description of AIDS. On the 30th anniversary, we defined priorities as improving use of existing interventions, clarifying optimal use of HIV testing and antiretroviral therapy for prevention and treatment, continuing research, and ensuring sustainability of the response. Despite scientific and programmatic progress, the end of AIDS is not in sight. Other major epidemics over the past decade have included Ebola, arbovirus infections, and coronavirus disease (COVID-19). A benchmark against which to compare other global interventions is the HIV/AIDS response in terms of funding, coordination, and solidarity. Lessons from Ebola and HIV/AIDS are pertinent to the COVID-19 response. The fifth decade of AIDS will have to position HIV/AIDS in the context of enhanced preparedness and capacity to respond to other potential pandemics and transnational health threats.

“When the history of AIDS and the global response is written, our most precious contribution may well be that, at a time of plague, we did not flee, we did not hide, we did not separate ourselves.”

—Jonathan Mann, Founding Director of Project SIDA and the World Health Organization Global Programme on AIDS, 1998

Forty years ago, on June 5, 1981, the Centers for Disease Control’s Morbidity and Mortality Weekly Report described 5 cases of *Pneumocystis* pneumonia in gay men (*1*). That report heralded the HIV/AIDS pandemic, which has resulted in over 75 million HIV infections and 32 million deaths. In 2011, we reviewed 30 years of AIDS and commented that the HIV/AIDS response would be a benchmark against which responses to other health threats would be compared (*2*). After 40 years of AIDS, we present our personal reflections on scientific and global health evolution over the fourth decade of AIDS in a world that has recently suffered other major epidemics. We focus on biomedical advances because these have had the greatest effect on HIV transmission and disease; advances in structural and behavioral interventions are reviewed in the CDC Compendium of Evidence-Based Interventions and Best Practices for HIV Prevention (*3*).

After the initial MMWR report was published, it took 2–3 years for the cause of AIDS, the novel retrovirus designated HIV, to be identified (*4*,*5*), and many more years to uncover its simian origin (*6*). Because of the asymptomatic spread of HIV, the long incubation period before disease, and transmission through sex and blood, millions of persons around the world, including several hundred thousand in the United States, were infected by the time the first AIDS cases were reported. The epidemiology and natural history of HIV infection, combining elements of acute and chronic diseases, ensured a diverse and long-lasting pandemic.

The history of HIV/AIDS and the struggle to contain it have seen the best and worst of human nature. Frequent examples of discrimination and exclusion are contrasted by leadership, illustrated by community activists (*7*), Jonathan Mann molding the first global response (*8*), Kofi Annan rallying the United Nations behind the search for a global fund (*9*), and President George W. Bush committing United States generosity to a war on HIV/AIDS of uncertain duration (*10*). Despite continued instances of injustice, the story has overall been a positive one, providing lessons for how to respond to other epidemic and pandemic threats.

## Evolving Epidemiology

The Joint United Nations Programme on HIV/AIDS (UNAIDS) estimates that in 2019, 38 million persons worldwide were living with HIV, 1.7 million became newly infected, and 690,000 died with HIV disease (*11*). Compared with 2010 estimates, overall HIV incidence in 2019 decreased by 23% and mortality by 37%. However, age stratification shows that new infections have decreased by 52% among children but by only 13% among adults. With reduced mortality rates yet continued HIV incidence and population growth, the overall number of persons living with HIV was 24% greater in 2019 than in 2010.

Global summaries hide regional differences. The epicenter of the pandemic remains in East and southern Africa, which account for 54% of all HIV-infected persons and 43% of incident HIV infections and deaths (*11*). High prevalence of HIV-infected persons with unsuppressed viremia predicts high incidence and maintenance of community infection, an observation that applies to regions and countries, as well as specific populations such as men who have sex with men (MSM).

The next greatest HIV burden is in the Asia and Pacific region, where the population is vastly greater than that of East and southern Africa but there are 3.5 times fewer HIV-infected persons (*11*). Despite overall prevention progress, HIV incidence has not declined equally everywhere; little success has been seen in eastern Europe, the Middle East, and Central Asia.

Ever clearer is the global burden of HIV in key populations: MSM, transgender persons, people who inject drugs, sex workers and their clients, and incarcerated persons. In 2019, an estimated 62% of all new HIV infections were in members of those key populations (*11*). In 7 of the 8 UNAIDS regions, key populations accounted for 60%–99% of incident HIV infections; only in East and southern Africa, where the proportion was 28%, were new infections predominant in general populations (*11*).

Among high-income nations, the most heavily affected country is still the United States. In 2018, a total of 37,881 HIV infections were newly reported, with regional differences (*12*). In the South, the rate of new infections was more than twice that for the Midwest, where the rate was the lowest. Major disparities by race/ethnicity persist; the rate among Black/African American persons is 2 times that among Hispanic and 8 times that among White persons. Also associated with higher rates are factors indicating social deprivation and poverty, even allowing for racial and ethnic disparities. Among new HIV infections, 70% resulted from male-to-male sex. A cause for concern is potential overlap between the HIV/AIDS and opioid epidemics through increased drug injection and needle sharing, which has resulted in explosive HIV outbreaks (*13*).

## Evolving Science and Program

In our 2011 commentary (*2*), we considered the following as priorities: improving use of existing interventions, defining how best to use HIV testing and antiretroviral therapy (ART) for prevention as well as treatment, continuing the quest for new knowledge and interventions, and ensuring sustainability of the global response. By and large, progress has been made on all fronts.

After the CAPRISA 004 trial of precoital and postcoital use of tenofovir gel was published in 2010 (*14*), the Ring (*15*) and Aspire (*16*) studies (randomized, placebo-controlled trials in South Africa) examined the protective efficacy of a self-inserted vaginal ring impregnated with slow-release dapivirine, a nonnucleoside reverse transcription inhibitor. The overall efficacy rates for reducing HIV incidence were 31% (Ring) and 27% (Aspire); many questions about overall efficacy, adherence, and differences by age remained. This collective experience provided proof of concept for woman-controlled prevention but did not provide the definitive public health solution to high HIV incidence among young women in Africa.

Four pivotal randomized trials (*17*–*20*) of oral preexposure prophylaxis (PrEP) with Truvada (combination of tenofovir and emtricitabine) were pivotal for international licensing of the compound. The relevant trials studied MSM, transgender women having sex with men, and at-risk heterosexual persons. A review of evidence considered another 9 studies, some of tenofovir alone, in different populations including people who inject drugs (*21*). The pivotal trials showed reduced HIV incidence (44%–86%) with Truvada use. However, a consistent observation has been a strong association between efficacy and adherence; PrEP is effective, but the drugs need to be taken.

Subsequent research focused on differential tissue penetration of drugs to relevant anatomic sites in men and women and on modes of drug delivery. HIV Prevention Trials Network (HPTN) studies compared the prevention efficacy of the long-acting injectable drug cabotegravir with Truvada in men and transgender women who have sex with men (study 083 [*22*]) and in heterosexual women (study 084 [*23*]). Interim results showed that cabotegravir, delivered every 8 weeks by injection, was associated with 66% lower incidence than oral Truvada in study 083 and 89% less in study 084. The long half-life of cabotegravir enables intermittent dosing, but waning drug levels over time may become subtherapeutic, thus requiring additional interventions to prevent infection and preclude development of drug resistance.

In its 2016 guidelines, the World Health Organization (WHO) recommended public health use of PrEP, as have other national and international regulatory or advisory bodies. However, the enthusiasm engendered by PreP science needs to be tempered by consideration of cost, need for rigorous adherence, rising rates of other sexually transmitted infections and thus need for continued condom use, and contraception for women. Long-acting injectables could be a major advance, but accessibility and logistics for their delivery need to be considered.

Mathematical modeling and ecologic studies suggested that greatly increased delivery of ART could reduce HIV transmission at the community level. The definitive study showing that ART provided prevention benefits was the landmark HPTN 052 study (*24*), published in interim form in 2011. This trial among discordant couples found a 96% reduction in HIV transmission among those who started ART early versus those for whom it was deferred. Combined with an influential modeling study (*25*) that suggested that regular HIV testing and immediate use of ART could suppress and perhaps ultimately eliminate HIV transmission, the results of HPTN 052 led to studies in East and southern Africa of the so-called test and treat intervention (*26*–*29*). These studies were community randomized evaluations of widespread HIV testing and immediate ART compared with standard care; the primary endpoint was HIV incidence. These large, expensive implementation science studies yielded rich information but did not lead to local HIV elimination. Of the 4 studies, 2 showed no significant incidence reduction and the other 2 showed 20%–30% reduction.

One of the reasons for the unexpectedly modest differences in HIV incidence between intervention and control communities in the test and treat study was changing global practice with regard to when to start ART. In 2015, results of the START (*30*) and TEMPRANO (*31*) trials showed unequivocally that immediate ART, irrespective of CD4+ lymphocyte count, resulted in reduced HIV-associated disease and death, ending more than 2 decades of argument about when to start treatment. WHO rapidly changed global recommendations to immediately start ART, one result of which was erosion of differences between intervention and control communities in the test and treat trials.

Although test and treat did not reduce HIV incidence to the extent hoped for, the accumulated evidence supports the notion of early, universal ART for extending the lives of HIV-positive persons as well as reducing the prevalence of unsuppressed viremia, the driver of HIV transmission. Large observational studies (*32*) showed that persons with suppressed viremia do not transmit the virus sexually, leading to the slogan “U = U”—undetectable equals untransmittable. This experience provides a much more compelling argument for active HIV case finding through increased HIV testing and partner notification, to enhance individual and public health through early treatment.

Although none of the approaches described provides a unique solution, the combination of widespread HIV testing, early ART for those infected, and PrEP for those at risk offers opportunity for substantially limiting the epidemic. Such approaches have been associated with reductions in new HIV infections among MSM in London, UK (*33*), and in New South Wales, Australia (*34*). In the United States, these advances—testing, case finding including through partner notification, universal treatment, PrEP, and rapid molecular investigation of clusters for service provision—have been incorporated into a revised national strategy for HIV elimination (*35*).

Progress toward an HIV vaccine remains discouraging. The only report of protective efficacy, published in 2009, has been the RV-144 study in Thailand (*36*), which investigated use of a recombinant canarypox vector vaccine (ALVAC-HIV) delivered in 4 monthly priming injections followed by a recombinant glycoprotein 120 subunit vaccine (AIDSVAX B/E) given in 2 additional injections. Reported efficacy was 26%–31%, but statistical and technical interpretation of these results was controversial (*37*). In 2016, the HVTN 702 study was launched in South Africa and used the same product as in the Thailand trial but modified for the dominant subtype C. After interim analysis, the study was halted for futility in early 2020 (*38*). Other efficacy studies of vaccines based on so-called mosaic immunogens from diverse HIV subtypes are in progress.

There has been great interest in broadly neutralizing antibodies to HIV, which some infected persons produce naturally and which might protect against a wide variety of strains. Two international trials of infusions with a broadly neutralizing antibody, VRC01, every 8 weeks showed relative protection against sensitive strains but no significantly reduced HIV incidence overall (*39*).

In 2014, UNAIDS launched its 90:90:90 initiative, aiming for 90% of persons with HIV infection to be diagnosed, 90% of those with an HIV diagnosis to receive ART, and 90% of those receiving treatment to show viral suppression by 2020. Globally, the respective proportions in 2019 were 81%, 82%, and 88%, so that an estimated 59% of persons living with HIV were showing viral suppression. Initially, 90:90:90 (with a goal of these numbers being 95s by 2030) was an advocacy proposal rather than an evidence-based initiative, but these targets have become adopted as policy promising “epidemic control,” itself a concept requiring precise definition (*40*).

ART scale-up, increased male circumcision, and prevention of mother-to-child transmission have all contributed to encouraging advances in the most heavily affected regions of Africa (*11*,*41*,*42*). Successful program implementation and declines in new HIV infections and deaths, combined with scientific progress, have led to a certain complacency that “AIDS is over.” Former US Secretary of State Hillary Clinton and staff promoted the idea that current tools could abruptly halt the epidemic. We largely agree with the 2018 judgment of the International AIDS Society–Lancet Commission on AIDS: “The HIV/AIDS community made a serious error by pursuing ‘the end of AIDS’ message” (*43*). Key populations, hiding in obscurity as well as in plain sight, will probably remain as reservoirs, even with highly performing programs. Experience in East and southern Africa has highlighted the challenge of adequate service provision to youth and men. Stigma and discrimination remain barriers in many parts of the world, and lack of an HIV cure (a priority research area) and vaccine remain scientific obstacles (*44*).

## Evolving Global Health

The HIV/AIDS pandemic has evolved in parallel with other global health events that necessarily influence how HIV/AIDS is perceived and prioritized. In a 2012 paper, author K.D.C. suggested that global health trends could best be analyzed through the lenses of development, public health, and health security (*45*). The fourth decade of AIDS started in the aftermath of the global financial crisis and the influenza (H1N1) pandemic and is finishing amid the coronavirus disease (COVID-19) pandemic. Although substantial progress has been made toward reducing maternal deaths, improving child survival rates, and scaling up programs for HIV/AIDS, malaria, and tuberculosis, the past decade has seen major disease outbreaks and a consequent focus on health security. Because of its sociodemographic effects, AIDS was portrayed as a security issue in United Nations discussions early in this century. With massive scale-up of treatment and prevention, HIV/AIDS is now perceived as another public health priority rather than a security emergency.

In 2014, Ebola was reported in Guinea, Liberia, and Sierra Leone, far west of previously recognized outbreaks. The epidemic lasted until mid-2016 and ultimately resulted in 28,646 reported cases and 11,323 deaths (*46*). Infections were exported to 3 other countries in Africa, several countries in Europe, and the United States. This health crisis resulted in widespread fear of possible global spread, unparalleled global mobilization of emergency health assistance including use of armed forces of the different high-income countries, and political involvement at the highest levels of governments and the United Nations. Subsequent outbreaks of Ebola have occurred in Uganda and the Democratic Republic of the Congo (DRC), including a large epidemic in conflict-ridden eastern DRC in 2018–2020 that resulted in 3,481 reported cases and 2,299 deaths (*47*). Underemphasized aspects of these Ebola epidemics were that cases over the past 6 years represent more than 90% of all cases reported cumulatively since recognition of Ebola in 1976; that vast geographic distances were involved; and that these outbreaks were largely urban, sometimes involving capital and other major cities. Ebola epidemiology has changed from that of an exotic, remote infection in Africa to one capable of causing extensive urban outbreaks threatening global health (*48*). Also of note was that field research conducted during the outbreaks under the most difficult conditions showed efficacy of a vaccine and therapeutics, both now considered the standard of care for Ebola (*49*,*50*).

Over the past decade, arboviral epidemic activity has been diverse. The epidemics of yellow fever in Angola and the DRC in 2015–2016 were the world’s largest over the past 30 years. A total of 965 cases and 400 deaths were reported, but true numbers were far greater. Over 30 million persons were vaccinated, and shortage of yellow fever vaccine required healthcare providers to resort to the untested practice of fractionating vaccine doses (*51*). Huge epidemics of chikungunya and dengue occurred internationally; virus was transmitted to areas previously considered at low risk, such as Europe (*52*). In 2015, the Zika epidemic raised global concern when infection with this virus was shown to be associated with microcephaly in infants and with Guillain-Barré syndrome and to be sexually transmissible. The outbreak resulted in at least 3,700 cases of birth defects in the Americas (*53*).

In 2005, after the outbreak of severe acute respiratory syndrome (SARS), WHO revised its International Health Regulations (*54*). A key change was authority to declare a Public Health Emergency of International Concern, a health emergency that could result in international spread or required coordinated action. WHO has implemented this authority only 6 times, 5 of them during the fourth decade of AIDS: for polio (2014), Ebola (2014 and 2019), Zika (2015), and COVID-19 (2019).

Related to health security are the interrelated challenges of global warming, demographic change, and migration. Climate change affects social and environmental determinants of health, such as access to clean air, water, shelter, and arable lands, but also exerts direct health effects. The United Nations High Commissioner for Refugees characterized 2010–2019 as “a decade of displacement,” during which 100 million persons were forced to flee their homes, many because of conflict such as that in the Middle East. During 2014–2020, some 20,000 migrants crossing the Mediterranean Sea to Europe drowned, and another 12,000 or more were unaccounted for.

Broad themes that have dominated global health discourse include the transition from the era of the Millennium Development Goals (MDGs; 2000–2015) to that of the broader Sustainable Development Goals (SDGs; 2015–2030) (*55*) and the issue of universal health coverage. Other disease-specific programs require continued support, such as the unfinished efforts to eradicate polio and Guinea worm disease. The MDGs had 3 specific health goals relating to child survival; maternal health; and HIV/AIDS, tuberculosis, and malaria. Only 1 of the 17 SDGs is devoted to health, SDG3, which has 13 targets and 28 indicators. Specifically, SDG3 calls for: “By 2030, end the epidemics of AIDS, tuberculosis, malaria and neglected tropical diseases and combat hepatitis, water-borne diseases and other communicable diseases.” Another target and WHO priority is provision of universal health coverage, global access to decent healthcare, and protection against penury from out-of-pocket health expenditures. HIV/AIDS exists in a crowded and complex global health space.

## Preparing for the Fifth Decade of AIDS

As the world emerged from the financial crisis a decade ago, there was concern that HIV/AIDS funding might be constrained. Development assistance for health reached $40.6 billion in 2019, an increase of 15% over the amount in 2010 (*56*). Approximately half of this assistance goes to HIV/AIDS, especially for treatment, and to newborn, maternal, and child health. Thus, although health security has eclipsed health development and global public health in this fourth decade of AIDS, financial commitments have been largely maintained.

The overall annual spending on HIV/AIDS by low- and middle-income countries is ≈$20.2 billion, of which ≈$9.5 billion represents donor funding. UNAIDS consistently communicates that to meet SDG targets, overall spending on HIV/AIDS needs to increase by ≈40%. Nonetheless, this HIV-specific spending is privileged compared with funding for other high-impact diseases in low-income settings, such as malaria and tuberculosis. AIDS is no longer among the 10 leading causes of death globally and is now widely viewed as a medically manageable disease. HIV/AIDS prioritization and funding may be justified by the youthful groups affected and its lifelong nature, but this view may be increasingly challenged. Expecting the United States to pay indefinitely for most of the world’s HIV/AIDS response is unrealistic. The end of the SDG era in 2030 will probably come with reappraisal of global commitments, including those for global health funding, disease-specific focus, and maintenance of single-disease organizations such as UNAIDS. Over the coming years, HIV/AIDS programs need to show good fiscal management and epidemiologic results, and affected countries need to shoulder an increased share of their disease burdens.

## Lessons from HIV/AIDS and Other Epidemics

The most dramatic epidemics in recent time (COVID-19 [*57*], Ebola, and HIV/AIDS) involve quite different biological agents and challenges yet also raise common themes and questions. Especially needed are global responses to challenges that transcend national borders. Pathogen emergence is enhanced by globalization, but globalized systems are needed to address an interconnected worldwide emergency. The slogan “no one is safe until everyone is safe” has been heard in relation to COVID-19, but it was said years ago about HIV. And global health needs global funding.

Individual leaders and organizations have performed valiant work on COVID-19, yet countries have isolated themselves in all senses, resulting in global fragmentation. Major powers look inward yet are reluctant to cede space, and the influence of multilateral agencies is limited. WHO was heavily criticized after the Ebola epidemic in West Africa but is constrained by restricted authority, inadequate funding, and unrealistic expectations from member states. Repeated calls for WHO reform are unclear about what is really wanted.

Honesty is required concerning preparedness and surveillance. The Ebola epidemic in West Africa became as severe as it did because the 3 affected countries had been neglected for years and had no functioning surveillance and public health infrastructure. We cannot say that severe acute respiratory syndrome coronavirus 2 (SARS-CoV-2) was completely unexpected; the literature on pandemic threats is voluminous. SARS in 2002–2003 was severe but not widespread; the 2009 influenza (H1N1) pandemic was widespread but not severe. It is hubristic to assume that pathogen severity and spread would always segregate, yet we were not prepared. Preparedness metrics can give false reassurance, witnessed by the lamentable response to COVID-19 in the United States in 2020. “Never again” was the mood after the Ebola epidemic in West Africa, but preparedness just seems too hard and costly. Perhaps true preparedness exists only in the military, where personnel train continuously for wars they hope will never happen.

As a result of technologic advances such as whole-genome sequencing, scientific progress on COVID-19 has been breathtakingly rapid compared with early laboratory research on HIV. We hope to not see a replay of the early history of ART, with scientific advances relating to COVID-19, and specifically vaccines, not being rapidly or equitably accessible everywhere. “Vaccine nationalism” is a new term raising the specter of lower risk groups in high-income countries receiving vaccine before, for example, frontline healthcare workers in low-income settings. Healthcare workers have been disproportionately affected by Ebola and COVID-19, highlighting the need for much greater investment in infection prevention and control in healthcare settings worldwide. Attention and innovation are required to ensure maintenance of HIV and other essential public health services amid other outbreaks such as COVID-19.

Although initially slow, the HIV/AIDS response over the years has been a beacon in global health for respect for individuals and their rights and for health equity. More reflection is required with regard to what the responses to HIV and Ebola have taught us and how they might be relevant to COVID-19 and other future epidemics.

## Conclusions

Although great need remains, the past decade has seen scientific and programmatic successes with regard to the HIV/AIDS priorities we defined after 30 years of AIDS. Existing interventions have been scaled up, and new tools such as PrEP and long-lasting drug preparations have been introduced. The roles of HIV testing and ART for treatment and prevention have been clarified, and the need for immediate ART for all HIV-infected persons has been proven. The global HIV/AIDS response has been sustained, financing has been maintained, and the world has kept focus on the SDGs. Mann’s judgment that “we did not separate ourselves” remains justified. We must also accept that political promises of “the end of AIDS” were hyperbole that current epidemiology does not support.

The COVID-19 pandemic has exploited the fault lines of global systems and existing inequalities in a way that HIV did early on. Regrettably, the solidarity that HIV/AIDS engendered has not yet been carried over. In retrospect, the recent epidemics of Ebola in West Africa and DRC were preparation for the COVID-19 pandemic, but follow-through was lacking. The fifth decade of AIDS will take us to the SDG target date and reassessment of global health and development priorities. HIV/AIDS may not be central to global health discourse as it was earlier, but it will remain a yardstick by which to judge commitment and efforts, including, and especially in relation to, health security.

## Addendum

On February 7, 2021, the Ministry of Health of DRC reported a laboratory-confirmed case of Ebola in North Kivu Province, the most heavily affected province during the 2018–2020 outbreak in eastern Congo. The case-patient experienced symptom onset on January 25, 2021, and died in Butembo, a city of ≈1 million persons, on February 4, 2021. She was reportedly linked epidemiologically to an Ebola survivor, and genetic sequencing reportedly showed phylogenetic association with the earlier outbreak rather than a new spillover event. As of February 8, 2021, a total of 118 contacts were being investigated (https://www.who.int/emergencies/diseases/ebola/ebola-2021-north-kivu, https://www.who.int/csr/don/10-february-2021-ebola-drc/en).

Separately, on February 14, 2021, the Ministry of Health of the Republic of Guinea reported an outbreak of Ebola in the subprefecture of Gouécké, Nzérékoré Region, the first report of Ebola in Guinea since the 2014–2016 epidemic. The index case-patient, a nurse, experienced symptoms on January 18, 2021, and died on January 28, 2021. A total of 6 secondary Ebola cases were reported, 1 in a traditional practitioner who cared for the index case-patient and 5 in family members attending her subsequent funeral. Of the 7 case-patients, 5 died. As of February 15, 2021, a total of 192 contacts were being investigated, including in the capital city, Conakry (https://www.who.int/emergencies/diseases/ebola/ebola-2021-nzerekore-guinea, https://www.who.int/csr/don/17-february-2021-ebola-gin/en).
